# Machine learning research based on diffusion tensor images to distinguish between anorexia nervosa and bulimia nervosa

**DOI:** 10.3389/fpsyt.2023.1326271

**Published:** 2024-01-11

**Authors:** Linli Zheng, Yu Wang, Jing Ma, Meiou Wang, Yang Liu, Jin Li, Tao Li, Lan Zhang

**Affiliations:** ^1^Mental Health Center, West China Hospital, Sichuan University, Chengdu, China; ^2^Affiliated Mental Health Centre and Hangzhou Seventh People's Hospital, Zhejiang University School of Medicine, Hangzhou, China

**Keywords:** anorexia nervosa, bulimia nervosa, eating disorder, DTI, machine learning

## Abstract

**Background:**

Anorexia nervosa (AN) and bulimia nervosa (BN), two subtypes of eating disorders, often present diagnostic challenges due to their overlapping symptoms. Machine learning has proven its capacity to improve group classification without requiring researchers to specify variables. The study aimed to distinguish between AN and BN using machine learning models based on diffusion tensor images (DTI).

**Methods:**

This is a cross-sectional study, drug-naive females diagnosed with anorexia nervosa (AN) and bulimia nervosa (BN) were included. Demographic data and DTI were collected for all patients. Features for machine learning included Fractional anisotropy (FA), axial diffusivity (AD), radial diffusivity (RD), and mean diffusivity (MD). Support vector machine was constructed by LIBSVM, MATLAB2013b, and FSL5.0.9 software.

**Results:**

A total of 58 female patients (24 AN, 34 BN) were included in this study. Statistical analysis revealed no significant differences in age, years of education, or course of illness between the two groups. AN patients had significantly lower BMI than BN patients. The AD model exhibited an area under the curve was 0.793 (accuracy: 75.86%, sensitivity: 66.67%, specificity: 88.23%), highlighting the left middle temporal gyrus (MTG_L) and the left superior temporal gyrus (STG_L) as differentiating brain regions. AN patients exhibited lower AD features in the STG_L and MTG_L than BN. Machine learning analysis indicated no significant differences in FA, MD, and RD values between AN and BN groups (*p* > 0.001).

**Conclusion:**

Machine learning based on DTI could effectively distinguish between AN and BN, with MTG_L and STG_L potentially serving as neuroimaging biomarkers.

## Introduction

Eating disorders (ED), comprising anorexia nervosa (AN) and bulimia nervosa (BN), are serious psychiatric disorders with severe physical health consequences and high mortality rates (1, 2). AN involves an enduring disregard for low body weight and dietary restrictions due to an intense fear of weight gain (3), while BN is characterized by recurrent episodes of binge eating and subsequent compensatory behaviors (4). Presently, the diagnostic process typically involves considering AN criteria before diagnosing BN. Notably, BN patients generally maintain a normal weight, yet the diagnostic boundaries between AN and BN remain unstable. A longitudinal study have indicated that approximately one-third of AN participants transition to BN (5), while the transition from BN to AN is less common but not absent (6). To sum up, distinguishing between AN and BN remains challenging.

Previous researchers have endeavored to differentiate AN from BN. A meta-analysis highlighted specific cognitive perspective impairments only in AN, not observed in BN (7). Furthermore, AN patients were found to exhibit deficits in low-level visuospatial processing and disturbance in the food-emotion relationship, unlike BN individuals, as suggested by neuropsychological task studies (8). Another study revealed that AN patients tend to employ emotion suppression as a maladaptive emotion regulation strategy, which differs from BN (9). Neuroimaging studies of AN and BN revealed some differences related to specific features of both disorders. For example, in a resting-state functional connectivity study found, women with AN showed stronger connectivity between the dorsal anterior cingulate cortex and retrosplenial cortex, while those with BN showed increased simultaneous activity between the dorsal anterior cingulate cortex and medial orbitofrontal cortex (10).

Machine learning, as a subfield of artificial intelligence, encompasses the utilization of heuristic algorithms for computers to acquire knowledge from data (11). Recently, In the pursuit of improved diagnosis and differentiation of mental illnesses, there has been a notable surge in the application of machine learning in brain imaging studies. The synergy between machine learning and brain imaging enables the analysis of vast neural data with increasing measurement precision (12). Lavagnino et al. (13) leveraged multivariate machine learning methods to analyze structural neuroanatomical scan data, emphasizing the potential of these methods for clinical translation at the individual subject level. Moreover, machine learning has demonstrated its capacity to enhance group classification without the need for researchers to specify variables (14). While still at an early stage in the study of ED, machine learning has emerged as an advanced computational too (15, 16). Cerasa et al. applied the support vector machine (SVM) technique to distinguish individuals with eating disorders from healthy controls (HCs), achieving a diagnostic accuracy of 0.80 in a small sample of ED vs. HCs (17). Additionally, machine learning has shown promise in predicting illness trajectories. In a longitudinal study by Haynos et al. (18), machine learning methods exhibited higher accuracy than traditional regression in predicting 2-year ED outcomes, potentially identifying crucial risk markers.

The investigation of brain structural alterations and function in eating disorders using diffusion tensor imaging (DTI) has started to yield valuable insights. Alterations in white matter microstructure have been observed in adolescents and adults with AN and BN. In a DTI study, adults and adolescent females with BN showed reduced partial anisotropy (FA) in white matter tracts extending through bilateral frontal and temporoparietal regions. Another DTI analysis revealed increased FA in the right corticospinal projection and lingual gyrus, along with significantly reduced FA in the corpus callosum, left superior longitudinal tract, and precentral gyrus in women with AN compared to healthy controls (19). Accumulating evidence suggests the presence of distinct differences in brain microstructure between AN and BN, although specific biomarkers for discrimination have not yet been identified.

To the best of our knowledge, few studies have combined machine learning with DTI to construct a model for distinguishing between AN and BN and to explore potential biomarkers. In this study, we attempted to employ machine learning methods to differentiate between drug-naïve females with AN and BN based on DTI data.

## Materials and methods

### Participants

A total of 58 drug-naive female patients, consisting of 24 AN and 34 BN, were enrolled in this study. All participants had not received systematic psychotherapy or physical therapy in the last year. Patients were diagnosed with DSM-5 disease by two psychiatrists using the Structured Clinical Interview for Diagnosis (SCID). Inclusion criteria required participants to have a diagnosis of AN or BN, between 14 and 50 years of age, female, and right-handed. None of the participants presented other major psychiatric disorders, head trauma, substance abuse or dependence, or neurological diseases.

All participants and guardians (if younger than 18 years) were fully informed of the details and purpose of the study and provided written informed consent before their participation. The study was approved by the West China Hospital of Sichuan University Biomedical Research Ethics Committee.

### Data acquisition

Data from all participants were collected using a Philips 3.0 T device. T1-weighted images and diffusion tensor images were acquired. The method of MRI data acquisition and scanning parameters have been described previously (20, 21). All scans were reviewed by a practicing neuroradiologist to exclude gross brain abnormalities.

### Image processing and feature extraction

All images were processed using FSL5.0.9 software, including head movement and eddy current correction and gradient direction correction. Subsequently, we selected the range of tensors that needed to be calculated according to the b0 image to obtain the FA, MD, AD, and RD images of each subject. By nonlinear registration, all of the FA, MD, AD, and RD images were transformed into Montreal Neurological Institute (MNI) space, and FMRIB58_FA was used as a template. Thereafter, the mean values of FA, MD, AD, and RD were extracted using JHU-ICBM-tracts-maxprob-thr25 as the brain map.

### Machine learning classification

The machine learning process was implemented in LIBSVM, MATLAB2013b, and FSL5.0.9 software. Machine learning analysis was performed to distinguish between AN and BN patients. For the machine learning analysis, we chose FA, MD, AD, and RD and a combination of four parameters.

### Feature selection

The image of each subject was registered to the corresponding template and smoothed with a 3 mm kernel. Two-sample *t*-tests were performed on smoothed images with *p* < 0.001 and using the FWE model to obtain the difference area. Taking each differential voxel as the center, the value of the voxel within a small ball with a 3 mm radius was averaged as the characteristic value.

### Classification

A leave-one-out cross-validation method was used to select one participant at a time as the test dataset, and the remaining participants formed the training dataset. Data were then normalized and mapped to 0–1. Feature filtering was performed to avoid overfitting caused by superfluous characteristic values. Each feature and the label had a correlation coefficient, and the features with the absolute correlation coefficient within the top 5% were retained as the training subject.

The linear kernel support vector machine (SVM) was used for training to obtain the trained model. The test dataset is predicted based on the trained model and the final value is the average of the accuracy, specificity and sensitivity for each training. Additionally, receiver operating characteristic (ROC) curves were obtained in this procedure. ROC was a statistical tool to describe the accuracy of a diagnostic test and was used to indicate the correlation between true positive and false positive rates. The area under the ROC curve (AUC) was an objective number that summarized the accuracy of the test. A test with an AUC of 1.0 has the perfect diagnostic ability, while an AUC of 0.5 means a test is not capable of discriminating between two conditions.

Finally, the contributing brain area was obtained from the feature location that contributed to prediction according to the trained model.

### Statistical analysis

Statistical analysis was performed using SPSS statistics version 20. Group comparisons for demographic data were performed using independent *t* tests for continuous variables, including age, BMI, years of education, and duration of disease. An analysis of variance, controlling for age, was employed to compare the differences in four aspects between AN and BN patients. The defined significance *p*-values for all statistical analyses were < 0.05.

## Results

### Clinical demographics

Twenty-four drug-naïve females with AN and 34 with BN were included in this study. There were no significant differences in age (*T* = 0.508, *p* = 0.613), years of education (*T* = 1.953, *p* = 0.056), or course of illness (*T* = 0.508, *p* = 0.613). AN patients had a much lower BMI than BN patients (*T* = 7.200, *p* < 0.001) (Table 1).

**Table 1:** Comparison of demographic data between AN and BN.

	AN (*N* = 24)	BN (*N* = 34)	*T*	*p* value
Age (year)	22.83 ± 4.12	23.50 ± 5.41	0.508	0.613
BMI (kg/m^2^)	15.78 ± 2.27	20.74 ± 2.78	7.200	**<0.001**
Education (year)	14.50 ± 2.75	15.85 ± 2.49	1.953	0.056
Duration of disease (year)	2.48 ± 1.78	2.91 ± 3.89	0.508	0.613

Data are presented as mean ± SD. AN, anorexia nervosa; BN, bulimia nervosa.Bold value indicates significant differences between AN and BN.This *p*-value means that the BMI of AN is significantly different from the BMI of BN patients.

### Machine learning results for the AN and BN groups

#### FA ML, MD ML, and RD ML model results

When FA, MD and RD were selected as data features, no significant differences were detected between the diffusion tensor images of subjects with AN or BN (voxel-level *p* > 0.001, cluster-level *p* > 0.05, FWE correction), indicating that it was difficult to discriminate via machine learning.

#### AD ML model results

**Figure 1:**
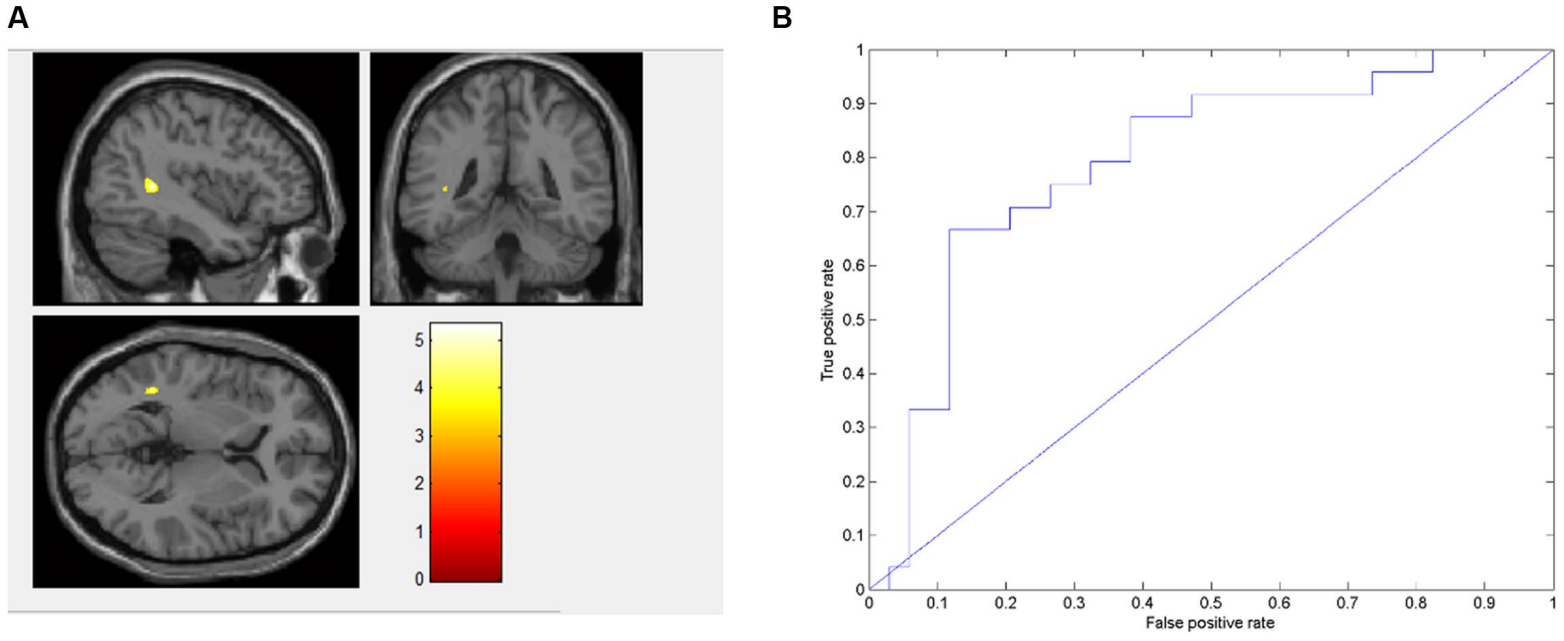
Presents the distinct brain regions in terms of AD value between AN and BN in **(A)**; the ROC curve of the AD machine learning model is depicted in **(B)**.

Using AD values as data features, AN patients exhibited lower value in the left middle temporal gyrus (MTG_L) and the left superior temporal gyrus (STG_L) than BN (Figure 1A). The model achieved an accuracy of 75.86%, a sensitivity of 66.67%, a specificity of 88.23% and an average area under the curve (AUC) of 0.793. The classification performance of this model was moderate (Figure 1B). AN patients exhibited lower AD features in the STG_L and MTG_L than BN.

## Discussion

To be the best of our knowledge, this is the first machine learning study to distinguish AN and BN based on DTI. In this study, AN patients exhibited lower AD value in MTG_L and STG_L than BN patients, with no significant difference between the two groups concerning FA, MD, and RD value. Furthermore, a machine learning model based on AD values of AN and BN patients showed that the AUC of the AD model was 0.793, with an accuracy of 75.86%, a sensitivity of 66.67%, and a specificity of 88.23%. The classification performance of this study is better than that of previous machine learning study studies on BN using other brain image parameters as data features.

The study showed AN patients had lower AD value in MTG_L compared to individuals with BN, which was consistent with a recent study indicating hyperactivity in the MTG of BN patients. In contrast, this brain area of AN patients demonstrated hypoactive (22). This might be attributable to the fact that abnormalities in the MTG are associated with physical dissatisfaction. A neuroimaging study found that AN patients have a volume reduction in the MTG, and its volume is correlated with body dissatisfaction (23). A functional magnetic resonance imaging study also demonstrated the MTG of AN patients exhibited notably diminished activation during self-other body size comparisons (23). Regarding the potential pathophysiological implications of our findings, it is noteworthy to mention that the MTG are associated with higher-order visual perception including the human body image. A notable augmentation in the neural activity within the MTG was observed among individuals diagnosed with ED subsequent to their participation in cognitive behavioral therapy targeting body image concerns (24). Compared with healthy controls, patients with AN showed significantly less activation of the middle temporal gyrus when comparing body image (25), which might be related to the key symptoms of AN, such as body image recognition disorder. In addition, we found that AN patients had lower AD value in STG_L compared to individuals with BN, which was in line with the previous research suggesting elevated activation of the STG in response to food image stimuli in patients with BN compared to AN patients (26). This could potentially be explained by heightened susceptibility of the STG to activation, which may contribute to the development of uncontrollable impulsive binge eating behavior in BN patients. The lower AD value might be related to the key symptoms of AN, such as restricted eating (27).

However, no significant differences were observed between AN and BN in the FA, MD and RD values. This might be attributed to the following two factors: First, the diagnostic crossover between AN and BN has been documented previously (5). Several longitudinal follow-up and retrospective studies have revealed that over time, a diagnosis of AN can evolve into a diagnosis of BN in 20 to 50% of patients (28–30). For instance, a study found that approximately 27% of patients initially diagnosed with BN eventually transitioned to a diagnosis of AN (31). Second, there were numerous shared behaviors between AN and BN, suggesting potential common biologic bases. The research has shown that both disorders exhibited structural connectivity abnormities in pathways from the orbitofrontal cortex and amygdala to the hypothalamus (32). A review also indicated that white matter alterations in AN are similar to BN in certain respects (32). Besides, several factors, notably the differing sensitivities of the metrics across distinct states, may underlie the significant difference found exclusively in AD rather than in FA, MD, and RD between AN and BN groups (33). AD might exhibit greater sensitivity to structural alterations or pertinent biological changes in the studied conditions, while FA, MD, and RD could be less sensitive or affected differently by the specific pathological processes examined. Further research is needed to elucidate the underlying mechanism.

The study had two key strengths. Firstly, it was the first study utilizing DTI images in a machine learning study for distinguishing between AN and BN. Secondly, all the patients were not under any medication, thereby minimizing the influence of drugs on the findings. The present study still has some limitations. Firstly, the sample size was relatively small. Secondly, the validation of the model’s performance would have been enhanced with the inclusion of an independent sample for testing. Thirdly, the study solely utilized diffusion tensor brain imaging of individuals with eating disorders as a data feature, which limited the diversity of the dataset. Fourthly, it’s imperative to consider further correction of the differential brain regions identified in our study, as the observed alterations in acute AN may have been influenced or biased by the partial volume effects (34, 35). Fifthly, AN patients, in contrast to BN, experience prolonged states of hunger. Previous studies have reported alterations in the hypothalamus, anterior cingulate and orbitofrontal cortex during hunger and satiety (36, 37). And the presence of active compensatory behaviors exhibited by BN patients before undergoing MRI scan might also contribute to differences observed in DTI images. Sixthly, the analysis did not include an assessment of the correlation between clinical characteristics and the distinct brain regions with observed differences. Consequently, it remains challenging to fully comprehend the relationship between specific brain regions and the pathology of the disease.

## Conclusion

In conclusion, the study demonstrated that despite overlapping clinical presentations, distinct patterns in AD values within the MTG_L and STG_L could serve as potential neuroimaging biomarkers for distinguishing between AN and BN. Further exploration and validation of these identified regions could significantly contribute to the refinement of diagnostic strategies and treatment interventions for these complex eating disorders.

## Data availability statement

The datasets presented in this study can be found in online repositories. The names of the repository/repositories and accession number(s) can be found in the article/supplementary material.

## Ethics statement

The studies involving humans were approved by the West China Hospital, Sichuan University. The studies were conducted in accordance with the local legislation and institutional requirements. Written informed consent for participation in this study was provided by the participants’ legal guardians/next of kin.

## Author contributions

LiZ: Writing – original draft, Writing – review & editing. YW: Writing – original draft, Writing – review & editing. JM: Data curation. MW: Data curation. YL: Data curation, Writing – review & editing. JL: Data curation, Writing – review & editing. TL: Data curation, Writing – review & editing. LaZ: Funding acquisition, Methodology, Writing – review & editing.
